# Acculturation moderates cognitive performance in adults at risk of autosomal dominant Alzheimer's Disease.

**DOI:** 10.1093/geroni/igab046.3659

**Published:** 2021-12-17

**Authors:** Kayla Tureson, Christopher Beam, John Ringman

**Affiliations:** 1 University of Southern California, Los Angeles, California, United States; 2 University of Southern California, University of Southern California, California, United States; 3 Keck School of Medicine at USC, Keck School of Medicine at USC, California, United States

## Abstract

Acculturative processes via persistent exposure to a set of cultural practices and behaviors can influence cognitive functioning (Park & Huang, 2010). The impact of acculturation on populations at risk for Alzheimer’s disease (AD), however, remains understudied. Persons with or at-risk for early-onset autosomal dominant AD (ADAD) offer a known AD pathogenesis and the opportunity to study whether acculturation moderates their cognitive performance. The present study used a latent variable model to test whether effects of latent cognitive ability on observable cognitive performance depend on acculturation. Participants included 119 adults with or at-risk for ADAD, the majority of whom were of Mexican origin with various levels of U.S. acculturation. Participants completed the Cognitive Abilities Screening Instrument (CASI) and the Acculturation Rating Scale for Mexican Americans-II (ARSMA-II). Confirmatory factor analysis was used to estimate a latent general cognitive ability factor from nine domains (e.g., attention, abstraction and judgment). The ARSMA-II was used to test whether factor loadings depended on level of acculturation, covarying for mutation status. Results revealed ARSMA-II scores nearly significantly moderated the effects of general cognitive ability on abstraction and judgment (λ = 0.20, SE = 0.11, p = .070). Individual differences in general cognitive ability at lower levels of acculturation likely predict lower abstraction and judgment performance. Cognitive assessments may not equally represent true cognitive ability in Mexican-Americans. Although the CASI was developed as a cross-cultural measure of cognitive functioning, caution should be exercised in inferring true cognitive functioning in Mexican-Americans who may not be acculturated to the U.S.

